# Transcriptome analysis reveals upregulation of immune response pathways at the invasive tumour front of metastatic seminoma germ cell tumours

**DOI:** 10.1038/s41416-021-01621-5

**Published:** 2022-01-12

**Authors:** Tim Nestler, Priya Dalvi, Friederike Haidl, Maike Wittersheim, Melanie von Brandenstein, Pia Paffenholz, Svenja Wagener-Ryczek, David Pfister, Ulrike Koitzsch, Martin Hellmich, Reinhard Buettner, Margarete Odenthal, Axel Heidenreich

**Affiliations:** 1 Department of Urology, University Hospital of Cologne, Cologne, Germany; 2 Department of Urology, Federal Armed Services Hospital Koblenz, Koblenz, Germany; 3 Institute of Pathology, University Hospital of Cologne, Cologne, Germany; 4 Institute of Medical Statistics and Computational Biology, University Hospital of Cologne, Cologne, Germany; 5 Center for Molecular Medicine, University of Cologne, Cologne, Germany; 6 Department of Urology, Medical Unveristiy of Vienna, Vienna, Austria

**Keywords:** Germ cell tumours, Molecular medicine

## Abstract

**Background:**

Testicular germ cell tumours (TGCTs) have a high metastasis rate. However, the mechanisms related to their invasion, progression and metastasis are unclear. Therefore, we investigated gene expression changes that might be linked to metastasis in seminomatous testicular germ cell tumour (STGCT) patients.

**Methods:**

Defined areas [invasive tumour front (TF) and tumour centre (TC)] of non-metastatic (with surveillance and recurrence-free follow-up >2 years) and metastatic STGCTs were collected separately using laser capture microdissection. The expression of 760 genes related to tumour progression and metastasis was analysed using nCounter technology and validated with quantitative real-time PCR and enzyme-linked immunosorbent assay.

**Results:**

Distinct gene expression patterns were observed in metastatic and non-metastatic seminomas with respect to both the TF and TC. Comprehensive pathway analysis showed enrichment of genes related to tumour functions such as inflammation, angiogenesis and metabolism at the TF compared to the TC. Remarkably, prominent inflammatory and cancer-related pathways, such as interleukin-6 (IL-6) signalling, integrin signalling and nuclear factor-κB signalling, were significantly upregulated in the TF of metastatic vs non-metastatic tumours.

**Conclusions:**

IL-6 signalling was the most significantly upregulated pathway in metastatic vs non-metastatic tumours and therefore could constitute a therapeutic target for future personalised therapy. In addition, this is the first study showing intra- and inter-tumour heterogeneity in STGCT.

## Background

Testicular germ cell tumours (TGCTs) are the most common tumours in young men between the ages of 15 and 35 years [1]. TGCTs are very likely to metastasise, and approximately 37% of patients already show metastases at initial diagnosis [2]. However, little is known about the molecular mechanisms resulting in tumour progression and the development of metastases in TGCTs.

TGCTs are grouped as seminomas and non-seminomatous tumours based on histomorphology. Seminomas appear as very homogeneous and uniform tumours often with apparent lymphatic infiltration, whereas non-seminomas are heterogeneous and encompass a variety of histological subtypes. Non-seminomatous tumours lack prominent lymphatic infiltration. In addition to their histomorphological differences, the two groups have distinct protein expression profiles. The immunohistochemical pattern of positive staining for the KIT receptor and the stem cell-associated transcription factor OCT4 is exclusive to seminomas [3].

Even though seminomas appear morphologically homogeneous, their biological behaviour is variable with the development of metastases in some tumours or chemoresistance with early relapse in others [4]. These differences can potentially be attributed to tumour heterogeneity, which has been shown to play a crucial role in metastatic spread and resistance to therapy in various other cancer types [5–7]. With regard to metastatic spread, previous studies have assessed the messenger RNA (mRNA) and microRNA (miRNA) expression profiles of seminomas to predict metastatic status [8, 9].

However, little is known about gene sets associated with TGCT invasion and metastasis. A few studies on other tumour types have identified a limited panel of genes related to invasion, progression or metastasis. These genes have been shown to be distinctly upregulated at the invasive tumour front (TF) compared to the tumour centre (TC) in metastatic cases. For example, in prostate cancer, RhoA and CXCR4 expression is increased at the TF in comparison to the TC and associated with poor tumour differentiation [10, 11]. Similarly, higher expression of the miRNA-17/92 cluster at the invasion front of colorectal cancer has been strongly linked to early metastatic progression [12]. However, systematic investigations with respect to seminomatous testicular germ cell tumours (STGCTs) are missing.

Therefore, we investigated regional gene expression differences in seminomas with the intention of expanding the understanding of processes leading to tumour metastasis. The invasive TF and vital TC regions of the primary tumour were separated and analysed to identify oncogenes and tumour suppressor genes driving tumour progression. We hypothesised that the genes related to invasion, progression and metastatic spread are distinctly differentially regulated at the invasive TF.

## Materials and methods

### Patient and tissue samples

Samples from a total of 35 patients with non-metastasised (clinical stage: cSI; *n* = 21) and metastasised (cSII/III; *n* = 14) pure seminomas at initial diagnosis were analysed. For each patient, the TF and TC regions were studied. The inclusion criteria were as follows: (1) pure seminoma; (2) inguinal orchiectomy between 2003 and 2017, (3A) cSI disease without adjuvant treatment and uneventful follow-up of at least 2 years and (3B) cSII/III disease treated with received inductive chemotherapy (no minimum follow-up required).

Primary testicular tumour tissue samples from each patient were histologically evaluated by experienced uropathologists (MW, RB) with regard to pT stage and lymphovascular invasion. The following areas were marked on haematoxylin and eosin (H&E)-stained slides: invasive TF, viable TC, areas of necrosis or fibrosis and tumour-free tissue. Medical records of all included patients were reviewed retrospectively for clinical characteristics and outcomes.

STGCT patients selected for the study were identified using the clinical database of the Department of Urology at the University Hospital of Cologne. All patients provided consent, the study complied with the Declaration of Helsinki, and local ethics committee approval was obtained (University Hospital of Cologne 17-427).

### Laser capture microdissection (LCM) and RNA extraction

Corresponding formalin-fixed, paraffin-embedded tissue of the previously marked areas of the TF and TC was dissected using an automated microtome (HM 355S; Thermo Scientific, Kalamazoo, MI, USA). Sections for LCM were cut to a thickness of 10 µm. The number of sections per patient subjected to LCM varied between 4 and 10 sections for the TF and TC, respectively. PTEN-coated MembraneSlide 1.0 (Zeiss, Göttingen, Germany) slides were used for LCM. Each step was performed with the utmost care following standard operating procedures to avoid any RNase contamination. The microtome, LCM and RNA processing workbenches were cleaned with RnaseZap (Ambion, Vilnius, Lithuania) and DEPC-H_2_O after processing each patient sample.

**Fig. 1:**
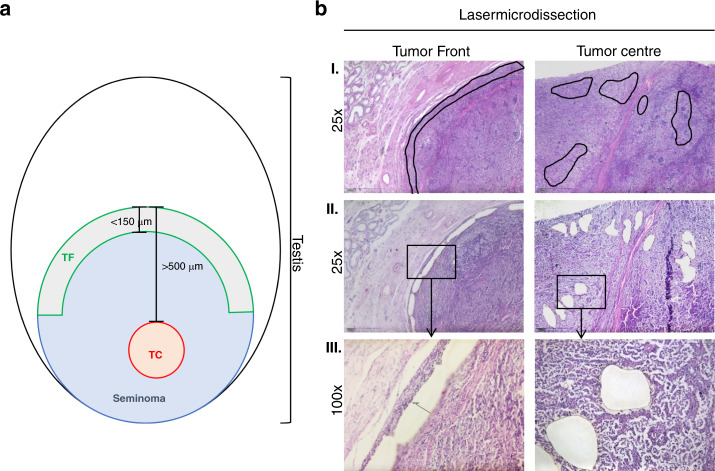
Definition of tumour areas for zonal transcriptional profiling. **a** Definition of the TF and TC tumour areas. **b** Representative histomorphological images depicting laser-microdissected TF and TC areas as defined above. Only viable tumour areas were dissected. I. Overview of the different areas: TF and TC at ×25 magnification with the marked areas prior to LCM. In rows II. and III., the dissected areas are shown at ×25 and ×100 magnification. TF tumour invasion front, TC tumour centre.

Immediately prior to LCM, the sections were deparaffinized and stained with Mayer’s haematoxylin (Haematoxylin cryst., Merck, Darmstadt, Germany) and 0.5% eosin (Waldeck, Münster, Germany) following the manufacturer’s instructions. The stained slides were covered with liquid cover glass (Zeiss, Göttingen, Germany) for better visualisation. The TF was defined as the region invading the normal tissue with a maximum distance of 150 μm from the invasive border of cancer, and the TC was defined as the area with a distance of at least 500 µm from the invasive border (Fig. 1) [13]. By using LCM, the TF and TC areas of each sample were collected separately (only viable tumour areas were dissected; areas with lymphatic infiltration were omitted). Tissue specimens were collected in AdhesiveCap 500 opaque tubes (Zeiss, Göttingen, Germany). All samples were incubated with proteinase K (Invitrogen, Carlsbad, CA, USA) and stored at −80 °C until further processing.

RNA isolation was performed for all samples using phenol–chloroform extraction and ethanol precipitation according to standard laboratory protocols. RNA samples were quantified using QuantiFluor® (Promega, Madison, WI, USA).

### NanoString mRNA profiling and normalisation

For NCounter-based gene expression profiling, RNA samples were diluted or concentrated using Savant DNA-SpeedVac-Concentrator DNA 120 (Thermo Scientific) to achieve the desired concentration of 200 ng/5 µl. In addition, every sample was spiked in with the positive and negative controls provided with the panel. This technique utilises capture and reporter probes specific for each gene labelled with a unique fluorescent barcode. Signals generated from these barcodes are automatically detected and quantified by the nCounter FLEX platform. The generated data were compiled as resource compiler (RCC) files.

RCC files were imported into nSolver 4.0 analysis software (NanoString Technologies, WA, Seattle, USA), and quality control steps were performed following the manufacturer’s guidelines. Raw data were processed sequentially: first background subtraction was performed using the formula: read count of each gene − 2 × (mean + 2 × SD) of all negative controls (eight genes). Next, the positive control normalisation factor was calculated according to the manufacturer’s instructions using positive controls that were spiked into every sample. Then, the CodeSet content normalisation factor (housekeeping normalisation factor) was calculated using reference genes to adjust for differences in analyte abundance and/or analyte quality across samples. The most stably expressed housekeeping genes (23 out of 30) were selected using the geNorm algorithm (Supplemental Table S1) [14]. Normalisation was performed per the default settings. The selection criteria for the positive control normalisation factor were 0.3–3.0. Considering these criteria, genes with a minimum of 40 read counts present in at least 25% of samples in either of the studied groups (687 out of 747 genes) were considered expressed and selected for further analysis.

### Bioinformatic analysis

To identify the different cellular processes represented by the expressed genes, the PANTHER classification system (v13.1) was used [15].

Genes with a false discovery rate <0.05 and a fold change (FC) > 1.5 were subjected to the upstream regulator and pathway analysis using the Ingenuity Pathway Analysis (IPA) platform (Qiagen, Redwood City, CA). IPA was performed for two groups: (1) the TF (cSI vs cSII/III) and (2) the TC (cSI vs cSII/III). The significance of the association between the genes from the dataset and the canonical pathways was determined based on two parameters: (1) a ratio was calculated as the number of genes from the dataset that mapped to a given pathway divided by the total number of molecules that make up the canonical pathway; (2) Fisher’s exact test was used to calculate a *p* value representing the probability that there is an association between the genes in the dataset and the canonical pathway that cannot be explained by chance alone. IPA upstream regulator analysis was used to identify potential transcriptional regulators that could explain the observed changes in gene expression between the metastasised and non-metastasised cases. The activation *z*-score was calculated to predict the activation or inhibition state of identified canonical pathways and transcriptional regulators based on published findings accessible through the ingenuity knowledge base. Further comparison analysis was performed on the TF (cSI vs cSII/III) and TC (cSI vs cSII/III) group datasets to determine differences in enriched functions.

Search Tool for the Retrieval of Interacting Genes/Proteins (STRING) analysis (v10.5) was performed to visualise and analyse different gene–gene interaction networks and the networks were explored with Cytoscape (v3.6) [16].

### Quantitative real-time PCR (qRT-PCR)

For qRT-PCR, we used RNA samples with excess after NanoString mRNA profiling (TF: cSI *n* = 7, cSII/III *n* = 4; TC cSI *n* = 8, cSII/III *n* = 5). Complementary DNA (cDNA) was obtained from 50 ng RNA using random primers and SuperScript III reverse transcriptase according to the manufacturer’s protocol (Invitrogen, Darmstadt, Germany). One microlitre of cDNA (transcribed from 50 ng RNA) was used for real-time PCR analysis. For quantitative analysis, *β-actin* was measured. All samples were normalised to *β-actin* as a reference gene. All experiments were done in duplicate. mRNA levels were determined according to the ∆∆CT method. The following primers were used: *β-actin*, forward: 5′-TTG GCA ATG AGC GGT TCC GCT G-3′ and reverse: 5′-TAC ACG TGT TTG CGG ATG TCC AC-3′; interleukin-6 (IL-6), forward: 5′-GCT ATG AAC TCC TTC TCC ACA AGC G-3′ and reverse: 5′-TGA AGA GGT GAG TGG CTG TC-3′.

### Enzyme-linked immunosorbent assay (ELISA) of serum samples

ELISA was used to assess the IL-6 concentration in serum samples of patients with cSI (*n* = 20) and cSII/III (*n* = 11) prior to orchiectomy. The commercially available IL-6 Human ELISA Kit (EH2IL-6) was applied using reagents supplied with the kit and following the manufacturer’s instructions (Thermo Fisher Scientific, Inc., Waltham, MA, USA). For ELISA, a FLUOstar Omega reader (BMG Labtech, Ortenberg, Germany) was used to detect absorbance at 450 and 550 nm, and the concentrations were calculated using a standard curve.

### Immunohistochemistry

Immunohistochemistry was performed using IL-6 antibody (mouse monoclonal; dilution 1:500 citrate buffer, Abcam, Netherlands) with a Bond Max automated system (Leica). The prerequisite for inclusion in the analysis was a homogeneous staining intensity. Semiquantitative assessment was applied for cytoplasmic staining considering 0 (negative, <25%), 1 (weak, 25–50%), 2 (moderate, 50–70%) and 3 (strong, >75%). In general, the highest staining intensity was reported.

To assess tumour-infiltrating lymphocytes (TILs), H&E-stained slides were used and recommendations of the International TILs Working Group 2014 were followed [17]. Briefly, TILs were evaluated for the stromal compartment within the borders of the invasive tumour, including monoclonal cells. TILs were assessed as a continuous parameter and grouped for further analysis to 0–10% stromal TILs, 20–40% and 50–90%.

### Statistical analysis

Statistical analysis was performed using the IBM SPSS Statistics system for Windows (v24.0) (Armonk, NY, USA) and GraphPad Prism 7 (GraphPad Software, La Jolla, CA, USA). Continuous variables are presented as the mean and standard deviation (SD), and categorical variables are presented as *n* (%). *T* tests or *χ*^2^ tests were used for group comparisons, and the Mann–Whitney *U* test was used for non-normal distributions. All statistical tests were two sided, with *p* ≤ 0.05 indicating significance.

## Results

### Patient characteristics

The studied cohort consisted of pure seminoma patients with either cSI disease without any adjuvant treatment or cSII/III seminoma patients with metastasis at initial diagnosis who were all treated with bleomycin/etoposide/cisplatin (BEP) for 3–4 cycles as inductive chemotherapy, depending on the International Germ Cell Cancer Collaborative Group (IGCCCG) risk classification (Table 1).

**Table 1:** Patient characteristics and clinicopathological features.

Characteristics	All patients	cSI	cSII/III	*P* value
*n*	35	21 (60.0)	14 (40.0)	
Age at diagnosis (years), mean ± SD	38.3 ± 9.0	38.6 ± 10.1	38.0 ± 7.5	0.857
Tumour size, mean ± SD	35.3 ± 25.2	29.1 ± 17.2	44.4 ± 32.6	0.079
Tumour size, *n* (%)				0.511
<4 cm	20 (57.1)	13 (61.9)	7 (50.0)	
>4 cm	15 (42.9)	8 (38.1)	7 (50.0)	
pT, *n* (%)				0.039
1	25 (71.4)	19 (90.5)	6 (42.9)	
2	10 (28.6)	2 (9.5)	8 (57.1)	
3	0	0	0	
4	0	0	0	
Invasion into lymphatic vessels, *n* (%)				0.028
0	29 (82.9)	20 (95.2)	9 (64.3)	
1	6 (17.1)	1 (4.8)	5 (35.7)	
Invasion into a vein, *n* (%)				0.056
0	32 (91.4)	21 (100)	11 (78.6)	
1	3 (8.6)	0 (0)	3 (21.4)	
cN, *n* (%)				<0.001
0	21 (60.0)	21 (100)	0	
1	0 (0)	0 (0)	0 (0)	
2	10 (28.6)	0	10 (71.4)	
3	4 (11.4)	0	4 (28.6)	
cM, *n* (%)				0.019
0	31 (88.6)	21 (100)	10 (71.4)	
1	4 (11.4)	0 (0)	4 (28.6)	
Clinical stage, *n* (%)				<0.001
I	23 (65.7)	23 (100)	0	
II	9 (25.7)	0	9 (64.3)	
III	5 (14.3)	0	5 (35.7)	
IGCCCG				—
Good	—	—	11 (78.6)	
Intermediate	—	—	3 (21.4)	
Serum marker				
AFP (<5.8 IU/ml), mean ± SD		2.7 ± 1.3	1.6 ± 0.7	
hCG (<5 mIU/ml), mean ± SD		1.0 ± 1.6	464.9 ± 1441.8	0.001
LDH (<250 U/l), mean ± SD		268.4 ± 104.2	569.3 ± 435.5	0.024
Treatment				<0.001
Chemotherapy	14	0	14	
3 × BEP	11	0	11	
4 × BEP	3	0	3	
Follow-up (months), mean ± SD	70.14 ± 52.6	59.8 ± 40.9	85.6 ± 65.0	0.342
Relapse	1	0 (0)	1 (7.1)	
Dead of disease	2	0	2 (14.3)	

*IGCCCG* International Germ Cell Cancer Collaborative Group, *AFP* alpha-fetoprotein, *hCG* human chorionic gonadotropin, *LDH* lactate dehydrogenase, *BEP* bleomycin, etoposide and cisplatinum chemotherapy.

For subgroups considering the status of metastasis (cSI vs cSII/III), group differences were calculated. IGCCCG risk classification is only defined for metastasised patients.

The baseline characteristics, such as age and tumour size, were not significantly different between the two patient groups with cSI disease and cSII/III (Table 1). However, the following tumour characteristics showed significant differences between the two groups: pathological tumour stage (pT), presence of lymphovascular invasion, serum concentrations of human chorionic gonadotropin, and lactate dehydrogenase levels prior to orchiectomy (Table 1). The serum alpha-fetoprotein levels were below the normal value (<5.8 IU/ml) in each patient. The mean follow-up intervals were not significantly different for cSI and cSII/III patients (59.8 ± 40.9 vs 85.6 ± 65.0 months; *p* = 0.342). In the cSII/III group, two patients died during chemotherapy (*n* = 1 tumour related, *n* = 1 therapy related), and one patient experienced relapse 33 months after initial chemotherapy.

### Gene expression analysis revealed the upregulation of immune pathways in cSII/III patients

**Fig. 2:**
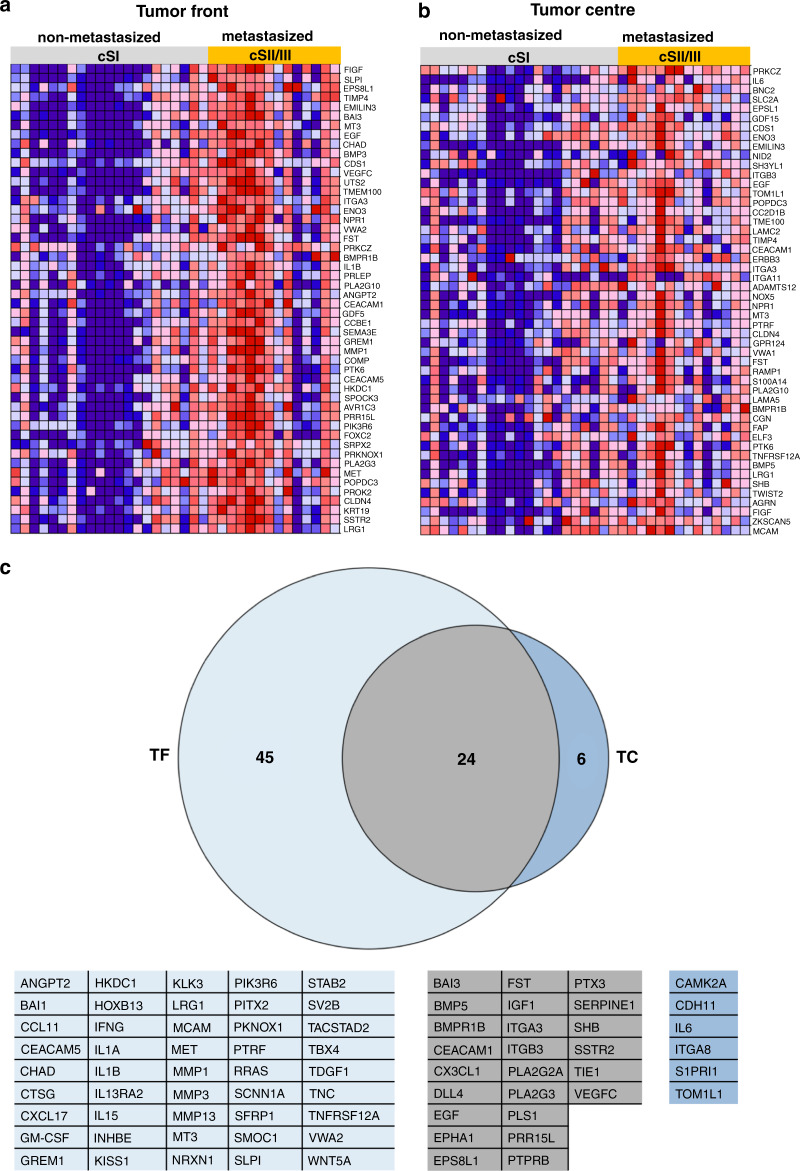
Differential expression of genes in metastatic vs non-metastatic tumours. Heatmaps representing the top 50 genes identified by gene set enrichment analysis (GSEA) to be significantly upregulated in metastasised (cSII/III) seminomatous cancer compared to non-metastasised (cSI). **a** The area of the invasive TF. **b** The corresponding TC area. Red indicates high gene expression, and blue indicates low gene expression. **c** Venn diagram shows genes related to immune processes that were significantly differentially expressed either in cSI vs cSII/III patients in the TF group (*n* = 27; marked slate blue) in cSI vs cSII/III patients in the TC group (*n* = 4; marked blue) or in both groups (*n* = 18; marked grey). The corresponding genes are listed below.

We investigated the differences in gene expression between non-metastasised and metastasised cases with respect to the TF and TC regions using the NanoString PanCancer Progression Panel, which includes a total of 770 genes related to cancer progression and metastatic spread. In cSII/III patients, compared to cSI patients, a total of 164 genes were upregulated and 2 genes were downregulated (FC ≥ ±1.5 and *p* ≤ 0.05) at the TF, whereas at the TC, only 85 genes were upregulated and 2 genes were downregulated. The plot of the top 50 differentially expressed genes in both the TF and TC groups showed a clear upregulation of these genes in cSII/III vs cSI patients, with a distinct expression pattern at the TF (Fig. 2a, b). The top 50 differentially expressed genes at the TF and TC in cSII/III vs cSI tumours were enriched for biological functions associated with metabolism, inflammation and angiogenesis.

IPA was performed to determine the enriched cellular pathways at the TF (cSII/III vs cSI) or TC (cSII/III vs cSI). Importantly, we identified 57 canonical pathways that were significantly and differentially regulated (*z*-score > ±2.0 and *p* < 0.05) in the TF and 20 that were significantly and differentially regulated in the TC (Supplemental Table S2A for TF and Supplemental Table S2B for TC). Of these, 16 in the TF and 4 in the TC were unequivocally linked to non-tumourous disease or different types of cancer. Of the remaining pathways, some were enriched only in the TF (*n* = 27), only in the TC (*n* = 2) or in both the TF and TC (*n* = 14). Most pathways represented by the differentially expressed genes were related to immune processes, the cell cycle, and angiogenesis. In addition, a functional comparison between both TF (cSII/III vs cSI) and TC (cSII/III vs cSI) groups showed enrichment of functions related to progression and metastatic spread, such as migration, invasion and angiogenesis, in the TF compared to the TC (Supplemental Fig. S1).

To identify the different cellular processes represented by the expressed genes, we subjected the initially selected 687 genes to PANTHER analysis. Interestingly, we observed that a large number of genes (*n* = 281) represented immune-related biological processes (Supplemental Table S3). As many canonical pathways were predicted to be related to immune processes and the largest subgroup of analysed genes showed immune processes as a biological function, we focused on these immune-related genes. Next, we analysed these 281 genes to determine the significantly upregulated genes (FC > ±2 and *p* < 0.05) between the TF and TC groups (cSI vs cSII/III, respectively) (Supplemental Table S4). We found 75 genes to be upregulated in either the TF or TC group; 69 were significantly upregulated in the TF area, while only 30 were significantly upregulated in the TC area (Fig. 2c).

### Comprehensive analysis showed the upregulation of IL-6 and integrin signalling in cSII/III patients

**Fig. 3:**
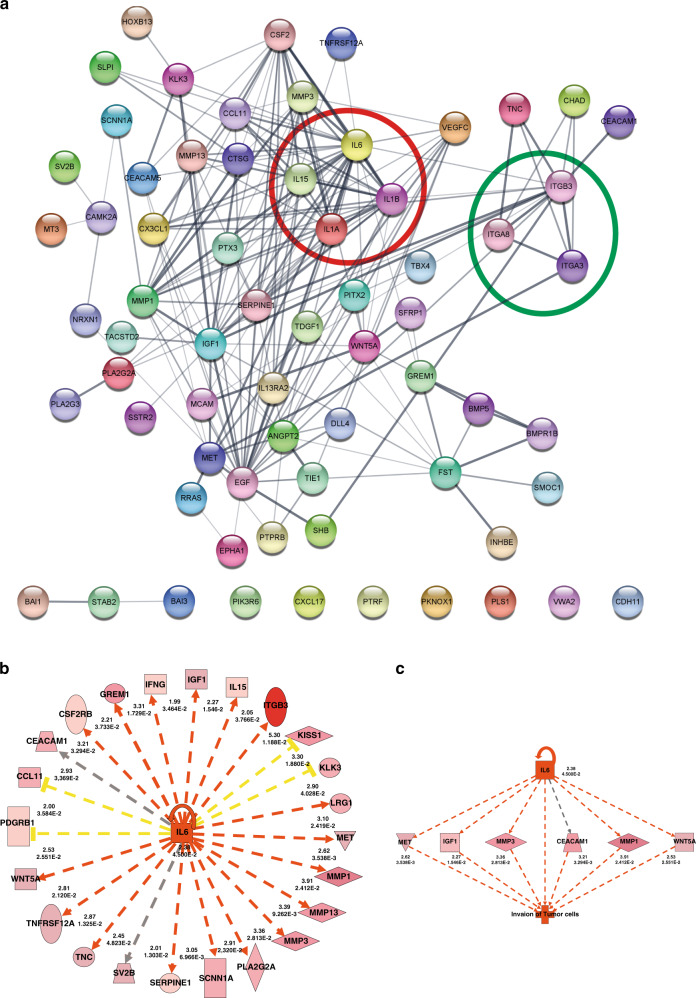
Network and upstream regulator analysis of IL-6. **a** Network interaction of the significant genes (FC > ±2.0 and *p* < 0.05) in cSI and cSII/III patients irrespective of localisation (TF and TC). Two distinct gene clusters were observed (red circle—interleukin cluster and green circle—integrin cluster). The network nodes represent proteins (encoded by the corresponding genes). **b** Ingenuity upstream regulator analysis predicted IL-6 as one of the top upstream regulators of gene expression in TF. The target genes of IL-6 identified in the dataset analysis are displayed in the figure. **c** Regulator effect network analysis by IPA identified IL-6 as an upstream regulator altering the expression of genes in the dataset that drive the invasion of tumour cells.

IPA was used to determine the pathways represented by the 75 differentially expressed genes in the TF or TC group (cSI vs. cSII/III, respectively). In the TC group, these genes did not represent any significantly enriched canonical pathways in cSII/III patients. However, in the TF, we noticed significant enrichment of 21 canonical pathways in the cSII/III patients compared to the cSI patients (Table 2A). Of particular importance, the IL-6 signalling pathway, represented by the genes *IL1A*, *IL1B*, *IL6*, *PIK3R6* and *RRAS*, was the most significantly upregulated in cSII/III vs cSI patients (Table 2B). Furthermore, we found that the integrin signalling pathway was significantly upregulated in cSII/III vs cSI patients (Table 2A). In addition, we performed STRING analysis with the same genes to identify relevant gene–gene interaction networks. Similar to the IPA results, STRING analysis showed that ILs and integrins were the most prominent gene networks (Fig. 3a).

**Table 2:** (A) Top 5 canonical pathways related to the immune system at TF, (B) dataset genes constituting the differentially expressed IL-6 pathway at TF and (C) top upstream regulators at TF predicted by IPA.

(A)				
Ingenuity Canonical Pathways	−Log (*p* value)	Ratio	*Z*-score	Molecules
IL-6 signalling	4.78E + 00	3.73E − 02	2236	IL1A, RRAS, PIK3R6, IL1B, IL6
Acute phase response signalling	4.23E + 00	2.86E − 02	2236	IL1A, RRAS, IL1B, IL6, SERPINE1
NF-κB signalling	4.12E + 00	2.72E − 02	2236	IL1A, RRAS, PIK3R6, IL1B, BMPR1B
Integrin signalling	2.79 E + 00	1.89E − 02	2	ITGA3, RRAS, ITGA8, PIK3R6
Fc epsilon RI signalling	3.65E + 00	3.20E − 02	2	RRAS, PIK3R6, PLA2G3, PLA2G2A

(B)						
Gene symbol	Entrez Gene Name	Expr fold change	Expr *p* value	Expected	Location	Type(s)
*IL6*	*Interleukin 6*	2.38	8.40E − 02	Up	Extracellular space	Cytokine
*IL1A*	*Interleukin 1 alpha*	3.52	1.90E − 02	Up	Extracellular space	Cytokine
*IL1B*	*Interleukin 1 beta*	2.27	7.00E − 03	Up	Extracellular space	Cytokine
*PIK3R6*	*Phosphoinositide-3-kinase regulatory subunit 6*	2.54	6.00E − 03	Up	Cytoplasm	Kinase
*RRAS*	*RAS related*	2.46	3.70E − 02	Up	Cytoplasm	Enzyme

(C)						
Upstream regulator	Fold change	Molecule type	Predicted activation state	Activation *z*-score	*P* value of overlap	Target molecules in dataset
IL1B	2.27	Cytokine	Activated	4.645	5.77E − 17	ADAM8, CCL11, CSF2RB, CX3CL1, DLL4, EDN1, ELF3, FST, HAS1, IFNG, IGF1, IL15, IL1A, IL1B, IL6, ITGB3, ITGB8, LAMA3, LAMC2, MMP1, MMP13, MMP3, PLA2G2A, PLA2G3, POSTN, PTGIS, PTX3, SCNN1A, SERPINE1, SLC2A1, SOX9, SSTR2, TIMP4, VEGF-C, WNT5A, ZC3H12A
NFkB	–	Complex	Activated	4.479	2.40E − 13	AQP1, BAD, CCL11, CX3CL1, EDN1, ELF3, FST, IFNG, IL13RA2, IL15, IL1A, IL1B, IL6, ITGB8, MMP1, MMP13, MMP3, MT3, PLA2G2A, PTX3, RRAS, SERPINE1, SLC2A1, SLPI, VEGF-C, WNT5A, ZC3H12A
IL1A	3.52	Cytokine	Activated	4.029	1.45E − 13	CCL11, IFNG, IGF1, IL1A, IL1B, IL6, ITGB3, JAM2, MCAM, MMP1, MMP13, MMP3, PTX3, SERPINE1, SSTR2, VEGF-C, WNT5A, ZC3H12A
VEGF	–	Group	Activated	3.882	3.83E − 10	ANGPT2, ANGPTL2, DLL4, EDN1, EMCN, ERBB3, FST, IFNG, IL1A, IL6, ITGB8, JAM2, MEOX2, MET, MMP13, NID2, SFRP1, TACSTD2, TBXA2R, TNC, VEGF-C
EGF	4.43	Growth factor	Activated	3.47	2.48E − 09	CLDN7, EDN1, ELF3, FST, FUT3, HRAS, IGF1, IL1B, IL6, ITGB3, KLK3, MET, MMP1, MMP13, MMP3, MT3, SERPINE1, TNC, TNFRSF12A, VEGF-C
PI3K	–	Complex	Activated	3,11	4.98E − 07	DLL4, HAS1, IFNG, IGF1, IL1B, IL6, MCAM, MMP1, MMP13, POSTN, PRKCZ, SERPINE1, SOX9
GM-CSF	2.21	Cytokine	Activated	3.011	1.08E − 05	ADAM8, BAD, CEACAM1, CSF2RB, EDN1, IFNG, IGF1, IL15, IL1A, IL1B, IL6, ITGB3, MET, MMP1
IL6	2.38	Cytokine	Activated	2.979	1.17E − 09	ADGRB1, CCL11, CEACAM1, CSF2RB, GREM1, IFNG, IGF1, IL15, IL6, ITGB3, KISS1, KLK3, LRG1, MET, MMP1, MMP13, MMP3, PLA2G2A, SCNN1A, SERPINE1, SV2B, TNC, TNFRSF12A, WNT5A
ANGPT2	2.35	Growth factor	Activated	2.566	7.87E − 04	EDN1, IL1B, IL6, MMP1, POSTN, SERPINE1, TNFRSF12A
MET	2.62	Kinase	Activated	2.213	1.70E − 04	EDN1, IL15, IL1B, IL6, ITGA3, MMP13
CCL11	2.93	Cytokine	Activated	2.211	9.11E − 05	CCL11, IL1B, IL6, MMP3, VEGF-C

FC > ±2.0, *z*-score ≥ 2.

### Upstream analysis predicted that tumour invasion is regulated by IL-6

Upstream pathway analysis was performed to determine the regulators mediating the differential expression of genes at the TF. Many enzymes (MET), cytokines (IL1B, IL1A, GM-CSF, IL6, CCL11) and growth factors (EGF, ANGPT2) were predicted to be upstream regulators of differential gene expression (Table 2C). Among these, IL-6 was one of the central upstream regulators that were predicted to be highly activated (*z*-score 2.98) and was also observed to be significantly upregulated at the expression level (FC 2.38, *p* < 0.001) in cSII/III vs cSI (Fig. 3b). Similarly, regulatory network analysis showed activation of the IL-6 network and, in response, the upregulation of its downstream targets. The IL-6 target genes *MET*, *IGF1*, *MMP3*, *CEACAM1*, *MMP1* and *WNT5A* were upregulated, which enabled the tumour cells to become invasive (Fig. 3c). Altogether, these findings demonstrate the significance of IL-6 expression as an upstream regulator of genes driving invasion predominantly in metastatic STGCT.

### Validation of IL-6 expression in the TF and TC

**Fig. 4:**
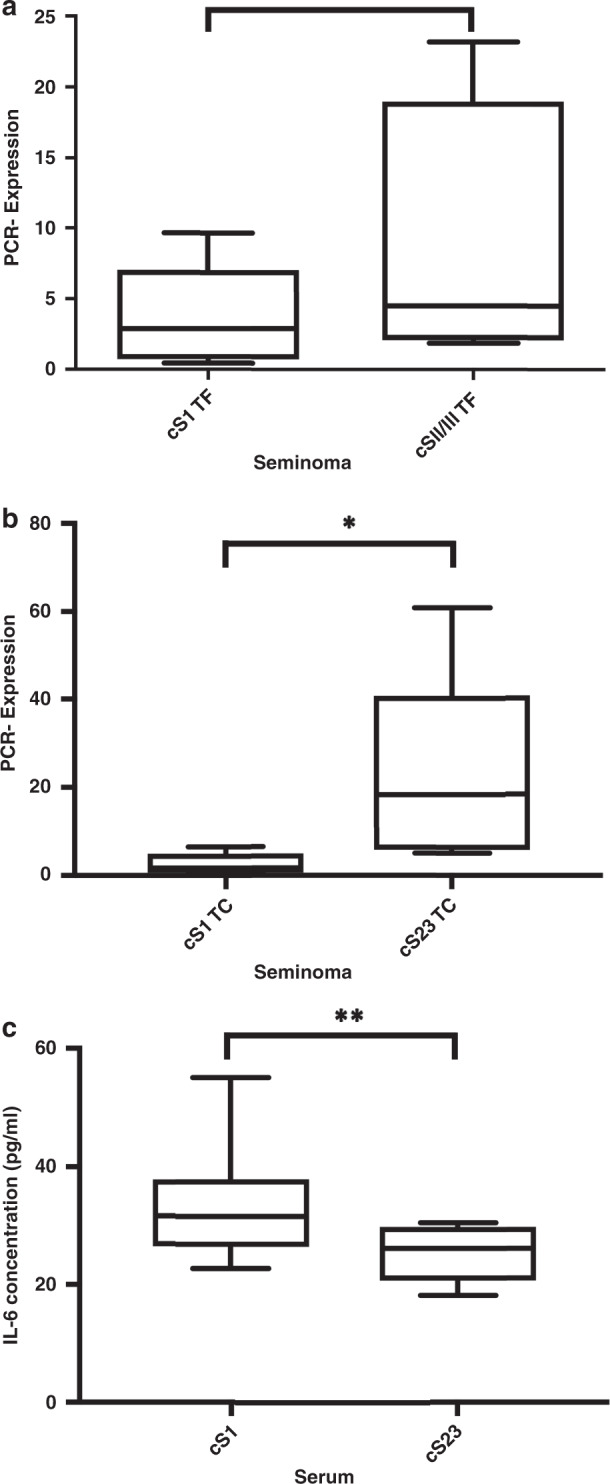
Validation of IL-6 using qRT-PCR of LCM samples and ELISA of serum samples. **a** PCR validation of IL-6 TF in cSI vs cSII/III patients in the TF and **b** TC. **c** IL-6 ELISA comparing cSI and cSII/III patients showed significantly elevated serum IL-6 levels in cSI patients. ^∗^*p* < 0.05 and ^∗∗^*p* < 0.01.

To validate the differential expression of IL-6 in seminomatous tissue with respect to the different tumour regions (TF and TC), we performed qRT-PCR. We compared the TFs of cSI vs cSII/III patients, and we found higher expression levels in cSII/III patients, but the difference was not significant (*p* = 0.278; Fig. 4a). However, for the TC group (cSI vs cSII/III), we found significantly higher IL-6 levels in metastatic cases (*p* = 0.028; Fig. 4b), as expected based on the NanoString data.

### ELISA of IL-6 in serum

In addition, we analysed IL-6 serum levels by comparing cSI and cSII/III patients. Interestingly, we found that the serum IL-6 level was significantly (*p* = 0.006) increased in the cSI group (Fig. 4c). These findings suggest that IL-6 might be less secreted in metastasised cases than in non-metastasised cases.

### Immunohistochemical analysis

IL-6 expression was assessed using immunohistochemistry for all patients studied by NanoString. IL-6 staining showed an intensive cytoplasmatic staining pattern in tumours of cSI and cSII/III patients with no significant group differences (*p* = 0.232; Supplemental Table S5 and Supplemental Fig. S2). TILs were assessed on the same cohort. Significantly more TILs were observed in seminomas of patients with cSI disease compared to those with cSII/III (*p* = 0.003; Supplemental Table S6 and Supplemental Fig. S3).

## Discussion

Tumour heterogeneity and intratumoural heterogeneity, in particular, represent a relevant challenge for personalised medicine, as taxonomically identical tumour entities show different clinical behaviours, resulting in tumour progression or therapeutic failure [18, 19]. In addition, in STGCT, tumour heterogeneity plays a significant role, as some tumours metastasise and several show occult metastasis in clinical stage I disease at the time of diagnosis.

With the aim of identifying tumour-driving forces leading to metastasis, we examined gene expression in different tumour regions (TF and TC) of metastasised and non-metastasised cases with seemingly uniform STGCT to achieve a better understanding of tumour biology and to improve diagnostic and therapeutic approaches.

We observed differential gene expression between the TF and TC regions of metastatic and non-metastatic cases. Interestingly, at the TF, almost double the number of genes was upregulated compared with the TC in metastatic versus non-metastatic tumours. This finding implies that the TF area is more active and displays an aggressive tumour phenotype in metastatic cases. These regional differences in gene expression represent inter- and intratumoural heterogeneity. Moreover, IPA showed significant enrichment of canonical pathways in the TF of metastasised cases. Pathways related to immune system processes were highly enriched, and similarly, gene ontology analysis revealed that most of the differentially expressed genes fell into the category of immune-related processes. Taken together, these results point to an important role of immune processes in the TF of metastatic tumours.

Further analysis of the immune-related genes showed that IL-6 was the most significantly divergent pathway at the TF in cSII/III vs cSI patients. In the validation analysis using PCR, IL-6 was slightly upregulated in cSII/III vs cSI patients. Likewise, IL-6 immunohistochemistry did not show a significantly different expression pattern between cSII/III vs cSI patients. It is probable that the IL-6 signalling pathway, which involves a group of different interacting genes, is upregulated at the TF rather than IL-6 as a single gene. Elevated levels of IL-6 have been shown to promote cancer cell proliferation, angiogenesis and metastasis [20]. Among TGCTs, IL-6 has previously been described to be significantly (*p* < 0.001) upregulated in seminomas and in germ cell neoplasia in situ compared to normal testicular tissue or testicular tissue with hypospermatogenesis [21, 22]. However, neither of the studies took into consideration the metastatic status of STGCTs. Using TCam-2 seminoma cells, Klein et al. demonstrated that an IL-6-enriched microenvironment is important for seminoma physiology [23].

Interestingly, we observed an inverse correlation of IL-6 levels in the seminomatous tissue and serum when comparing cSI and II/III patients. While IL-6 was overexpressed in the seminomas of cSII/III patients compared to cSI patients, the opposite pattern was found in the serum of these patients. This might be due to a decrease in IL-6 secretion during the process of tumour progression. In contrast, in other tumours, high expression levels of IL-6 have been correlated with impaired survival in triple-negative breast cancer, ovarian cancer and colorectal cancer and have been detected in the serum of metastatic prostate cancer patients [24–27]. These findings strongly indicate that IL-6 is of high significance for STGCT, but its functional relevance in relation to metastatic spread and the cellular mechanisms of IL-6 secretion has to be further examined.

IPA predicted that a regulatory network involving IL-6 and leading to the invasion of tumour cells was significantly activated. The genes observed in this network (*CEACAM1*, *IGF1*, *MET*, *MMP1*, *MMP3* and *WNT5A*) were upregulated in cSII/III and are known to be linked to invasive properties in several other cancer types [28–33]. However, this regulatory network has not been described for TGCTs, and the interaction of IL-6 with one of the mentioned downstream targets has not been further investigated for TGCTs. IL-6 has been attributed to invasive tumour function in pancreatic, bladder and gastric cancer [34–36]. IL-6 is a key player in metastatic spread and angiogenesis and acts in combination with other tumorigenic genes, such as *GM-CSF*, *VEGF* or *EGF* [37–41]; these three related genes were also upregulated in cSII/III STGCTs in our cohort. A recent study on colorectal cancer reported the release of IL-6 and GM-CSF from cancer-associated fibroblasts, leading to the differentiation of monocytes into tumour-associated macrophages, resulting in an immunosuppressive tumour niche. Furthermore, the upregulation of IL-6 and GM-CSF was observed in colon cancer tissue versus normal tissue according to the Oncomine database. Interestingly, the simultaneous targeting of both genes resulted in the inhibition of metastasis [37]. We also confirmed that in samples from metastatic cases, both genes were significantly more highly expressed than in non-metastatic cases. Another study showed the upregulation of IL-6, IL-1b and VEGF in pancreatic ductal adenocarcinomas in the presence of high concentrations of G-CSF [38]. The activation of VEGF-C by IL-6 was reported in other studies to result in lymphangiogenesis [39, 40]. Our finding of VEGF-C upregulation in cSII/III patients in the TF and TC is consistent with these data.

These findings are in accordance with our data that showed that EGF, GM-CSF and VEGF are upregulated in metastatic tumours and, along with IL-6, are predicted to be upstream regulators according to IPA. As another immuno-oncological approach, we analysed TILs in testicular tumours and demonstrated a correlation with non-metastatic disease. This finding is in line with recent publications, which also showed that TILs were correlated with metastatic stage and a low risk of recurrence pointing to possible prognostic relevance [42, 43].

Similar to the findings for IL-6, the integrin signalling pathway was also enriched at the TF of cSII/III patients. The expression of integrins is altered in cancer, and the integrin subunits affected depend on the type of carcinoma. Recent studies have shown that integrins have a crucial role in the progression and metastatic spread in different tumour entities [44, 45] and some studies have suggested a potential role in seminomas. Two studies have reported high immunohistochemical expression of integrin A6 in seminomas compared to normal testicular tissue [46, 47]. Timmer et al. compared the expression levels of different integrin subunits in metastasised and non-metastasised seminomas, but no compelling differences were observed [47]. Here, for the first time, we report higher expression levels of integrins (A3, A8 and B3) in metastasised seminomas than in non-metastasised seminomas.

Integrin B3, a subunit associated with early metastatic spread in breast cancer [48], was the most significantly upregulated integrin in our cohort of metastatic seminoma versus non-metastatic seminoma patients. These findings point to integrins as crucial players in the development of metastasis.

These findings demonstrate that conventional histopathological examination of ablated testicular tumours is not sufficient to understand complex and heterogeneous tumour biology. This is the first study highlighting the importance of IL-6 signalling and the integrin pathway for metastatic spread and identifying them as possible therapeutic targets in patients with chemotherapy resistance. In addition, we provide the first insights into the tumour heterogeneity of morphologically uniform seminomas. Validation of these findings in a larger cohort using different techniques, including immunohistochemical analysis, is underway.

Supplemental information is available at the *British Journal of Cancer* website.

## Supplementary information

Reproducibility Checklist

SUPPLEMENTAL MATERIAL

## Data Availability

Raw data are available from the authors upon request.
