# Supplementary material for: Transcriptome analysis reveals upregulation of immune response pathways at the invasive tumour front of metastatic seminoma germ cell tumours
Source: Br J Cancer. 2022 Jan 12;126(6):937–47. doi: 10.1038/s41416-021-01621-5 (PMC8927344; doi:10.1038/s41416-021-01621-5)

**Transcriptome analysis of metastatic and non-metastatic seminoma germ cell tumours reveals upregulation of immune response pathways at the invasive tumour front**

Tim Nestler^1,2^, Priya Dalvi^3,^ Friederike Haidl^1^, Maike Wittersheim^3^, Pia Paffenholz^1^, Svenja Wagener-Ryczek^3^, David Pfister^1^, Ulrike Koitzsch^3^, Martin Hellmich^4^, Reinhard Buettner^3^, Margarete Odenthal^3^, Axel Heidenreich^1^

^1^ Department of Urology, University Hospital of Cologne, Cologne, Germany

^2^ Department of Urology, Federal Armed Services Hospital Koblenz, Koblenz, Germany

^3^ Department of Pathology, University Hospital of Cologne, Cologne, Germany

^4^ Institute of Medical Statistics and Computational Biology, University Hospital of Cologne, Cologne, Germany

# Supplemental

**Table S1: List of housekeeping genes used in the geNorm algorithm** (14) **for normalization.**

| **Gene Name** | **Gene** | **Order selected by geNorm** | **SD after normalization** |
| --- | --- | --- | --- |
| FCF1-mRNA | NM_015962.4:1022 | 1 | 0,28 |
| PRPF38A-mRNA | NM_032864.3:335 | 2 | 0,246 |
| TRIM39-mRNA | NM_021253.3:3140 | 3 | 0,269 |
| DDX50-mRNA | NM_024045.1:1185 | 4 | 0,213 |
| GPATCH3-mRNA | NM_022078.2:1685 | 5 | 0,273 |
| TMUB2-mRNA | NM_024107.2:1485 | 6 | 0,294 |
| COG7-mRNA | NM_153603.3:1492 | 7 | 0,254 |
| HDAC3-mRNA | NM_003883.2:1455 | 8 | 0,239 |
| TLK2-mRNA | NM_006852.2:2335 | 9 | 0,283 |
| MRPS5-mRNA | NM_031902.3:390 | 10 | 0,279 |
| AMMECR1L-mRNA | NM_001199140.1:3564 | 11 | 0,268 |
| EIF2B4-mRNA | NM_172195.3:1390 | 12 | 0,273 |
| ERCC3-mRNA | NM_000122.1:1950 | 13 | 0,279 |
| CNOT10-mRNA | NM_001256741.1:1962 | 14 | 0,341 |
| USP39-mRNA | NM_001256725.1:806 | 15 | 0,33 |
| DHX16-mRNA | NM_001164239.1:2490 | 16 | 0,287 |
| SF3A3-mRNA | NM_006802.2:2060 | 17 | 0,308 |
| MTMR14-mRNA | NM_022485.3:720 | 18 | 0,309 |
| ZNF143-mRNA | NM_003442.5:925 | 19 | 0,298 |
| SAP130-mRNA | NM_024545.3:3090 | 20 | 0,313 |
| EDC3-mRNA | NM_001142443.1:925 | 21 | 0,335 |
| ZC3H14-mRNA | NM_001160103.1:2690 | 22 | 0,341 |
| CNOT4-mRNA | NM_001190848.1:795 | 23 | 0,346 |
| CC2D1B-mRNA | NM_032449.2:4182 | Not included in analysis | 0,556 |
| NOL7-mRNA | NM_016167.3:335 | Not included in analysis | 0,466 |
| ZKSCAN5-mRNA | NM_014569.3:3688 | Not included in analysis | 0,409 |
| DNAJC14-mRNA | NM_032364.5:1166 | Not included in analysis | 0,413 |
| AGK-mRNA | NM_018238.3:816 | Not included in analysis | 0,41 |
| NUBP1-mRNA | NM_001278506.1:304 | Not included in analysis | 0,471 |
| ZNF346-mRNA | NM_012279.2:2260 | Not included in analysis | 0,448 |

**Table 2SA: Analysis of significantly regulated canonical pathways (z-score >±2.0 and p<0.05) at TF (cSII/III vs cSI) using IPA predicts a total of 57 pathways of which n=41 are linked to tumour related or immune processes.**

| **Ingenuity Canonical Pathways TF cS23vs1** | **p-value** | **Ratio** | **z-score** | **Molecules** |
| --- | --- | --- | --- | --- |
| Actin Cytoskeleton Signaling | 0,0046 | 3,08E-02 | 2,449 | ITGA3,RRAS,FGFR4,PIK3R6,HRAS,EGF,APC |
| *Adrenomedullin signaling pathway | 0,0019 | 3,59E-02 | 2,646 | IL1A,BAD,RRAS,FGFR4,PIK3R6,HRAS,IL1B |
| *Agrin Interactions at Neuromuscular Junction | 0,0005 | 6,85E-02 | 2,236 | ITGA3,RRAS,HRAS,ERBB3,ITGB3 |
| Antioxidant Action of Vitamin C | 0,0025 | 4,81E-02 | -2 | CSF2RB,SLC2A1,PLA2G10,PLA2G3,PLA2G2A |
| Antiproliferative Role of Somatostatin Receptor 2 | 0,0007 | 6,41E-02 | 2,236 | SSTR2,RRAS,FGFR4,PIK3R6,HRAS |
| *Cardiac Hypertrophy Signaling | 0,0209 | 2,52E-02 | 2,449 | IGF1,MAPKAPK3,RRAS,FGFR4,PIK3R6,HRAS |
| ‡CCR3 Signaling in Eosinophils | 0,0000 | 7,58E-02 | 2,53 | RRAS,FGFR4,PLA2G10,PIK3R6,HRAS,PLA2G3,CCL11,PLA2G2A,PRKCZ,PRKCB |
| *CNTF Signaling | 0,0030 | 6,06E-02 | 2 | RRAS,FGFR4,PIK3R6,HRAS |
| *Colorectal Cancer Metastasis Signaling | 0,0000 | 7,63E-02 | 2,524 | IFNG,BAD,MMP3,RRAS,VEGFC,HRAS,MMP10,MMP13,EGF,APC,FGFR4,DCC,PIK3R6,VEGFD,NOS2,MMP1,WNT5B,MMP17,WNT5A |
| *Dendritic Cell Maturation | 0,0263 | 2,70E-02 | 2,236 | IL1A,FGFR4,IL15,PIK3R6,IL1B |
| EGF Signaling | 0,0033 | 5,88E-02 | 2 | FGFR4,PIK3R6,HRAS,EGF |
| Eicosanoid Signaling | 0,0003 | 7,69E-02 | 2 | PTGIS,PLA2G10,TBXA2R,PLA2G3,PLA2G2A |
| ‡Endothelin-1 Signaling | 0,0000 | 5,73E-02 | 2,111 | EDN1,RRAS,FGFR4,PLA2G10,PIK3R6,HRAS,PLA2G3,NOS2,PLA2G2A,PRKCZ,PRKCB |
| ‡ERK/MAPK Signaling | 0,0000 | 5,42E-02 | 2,111 | ITGA3,ELF3,BAD,RRAS,FGFR4,PLA2G10,PIK3R6,HRAS,PLA2G3,PLA2G2A,PRKCB |
| *Estrogen-Dependent Breast Cancer Signaling | 0,0010 | 5,88E-02 | 2,236 | IGF1,RRAS,FGFR4,PIK3R6,HRAS |
| ‡Fc Epsilon RI Signaling | 0,0000 | 7,20E-02 | 2,333 | RRAS,FGFR4,PLA2G10,PIK3R6,HRAS,PLA2G3,PLA2G2A,PRKCZ,PRKCB |
| FcγRIIB Signaling in B Lymphocytes | 0,0072 | 4,71E-02 | 2 | RRAS,FGFR4,PIK3R6,HRAS |
| FGF Signaling | 0,0091 | 4,40E-02 | 2 | MET,FGFR4,PIK3R6,HRAS |
| *GDNF Family Ligand-Receptor Interactions | 0,0065 | 4,88E-02 | 2 | RRAS,FGFR4,PIK3R6,HRAS |
| *Glioblastoma Multiforme Signaling | 0,0000 | 5,39E-02 | 2,333 | IGF1,RRAS,FGFR4,PIK3R6,HRAS,EGF,APC,WNT5B,WNT5A |
| Gα12/13 Signaling | 0,0089 | 3,55E-02 | 2,236 | RRAS,FGFR4,TBXA2R,PIK3R6,HRAS |
| ‡HGF Signaling | 0,0000 | 7,63E-02 | 2,121 | MET,ITGA3,ELF3,RRAS,FGFR4,PIK3R6,HRAS,PRKCZ,PRKCB |
| HMGB1 Signaling | 0,0000 | 5,84E-02 | 2,646 | IFNG,IL1A,RRAS,FGFR4,PIK3R6,HRAS,IL1B,SERPINE1 |
| IL-2 Signaling | 0,0036 | 5,71E-02 | 2 | RRAS,FGFR4,PIK3R6,HRAS |
| **IL-6 Signaling** | **0,0013** | **4,48E-02** | **2,449** | **IL1A,RRAS,FGFR4,PIK3R6,HRAS,IL1B** |
| ‡IL-8 Signaling | 0,0000 | 5,45E-02 | 2,714 | ANGPT2,RRAS,FGFR4,PIK3R6,VEGFC,HRAS,VEGFD,EGF,PRKCZ,ITGB3,PRKCB |
| ILK Signaling | 0,0001 | 4,66E-02 | 2,121 | FGFR4,PIK3R6,VEGFC,VEGFD,ITGB4,ITGB8,ITGB6,NOS2,ITGB3 |
| **Integrin Signaling** | **0,0000** | **5,19E-02** | **2,53** | **ITGA3,RRAS,TSPAN1,FGFR4,PIK3R6,HRAS,ITGB4,ITGB8,ITGB6,ITGA7,ITGB3** |
| JAK/Stat Signaling | 0,0085 | 4,49E-02 | 2 | RRAS,FGFR4,PIK3R6,HRAS |
| *Melanocyte Development and Pigmentation Signaling | 0,0145 | 3,85E-02 | 2 | RRAS,FGFR4,PIK3R6,HRAS |
| Mouse Embryonic Stem Cell Pluripotency | 0,0033 | 4,50E-02 | 2,236 | RRAS,FGFR4,PIK3R6,HRAS,APC |
| *Neurotrophin/TRK Signaling | 0,0009 | 6,02E-02 | 2,236 | KLK3,RRAS,FGFR4,PIK3R6,HRAS |
| ‡NF-κB Activation by Viruses | 0,0000 | 8,60E-02 | 2,121 | ITGA3,RRAS,FGFR4,PIK3R6,HRAS,PRKCZ,ITGB3,PRKCB |
| ‡NF-κB Signaling | 0,0000 | 5,43E-02 | 2,53 | IL1A,RRAS,FGFR4,PIK3R6,HRAS,IL1B,EGF,BMPR1B,PRKCZ,PRKCB |
| *NGF Signaling | 0,0052 | 4,03E-02 | 2,236 | RRAS,FGFR4,PIK3R6,HRAS,PRKCZ |
| Oncostatin M Signaling | 0,0000 | 1,25E-01 | 2,236 | MMP3,RRAS,HRAS,MMP13,MMP1 |
| *‡Ovarian Cancer Signaling | 0,0000 | 7,38E-02 | 2,828 | EDN1,RRAS,FGFR4,PIK3R6,VEGFC,HRAS,VEGFD,EGF,APC,WNT5B,WNT5A |
| p38 MAPK Signaling | 0,0008 | 0,05 | 2,449 | IL1A,MAPKAPK3,PLA2G10,PLA2G3,IL1B,PLA2G2A |
| PAK Signaling | 0,0027 | 4,72E-02 | 2,236 | ITGA3,RRAS,FGFR4,PIK3R6,HRAS |
| Paxillin Signaling | 0,0000 | 8,62E-02 | 2,236 | ITGA3,RRAS,FGFR4,PIK3R6,HRAS,ITGB4,ITGB8,ITGB6,ITGA7,ITGB3 |
| PEDF Signaling | 0,0100 | 4,30E-02 | 2 | RRAS,FGFR4,PIK3R6,HRAS |
| ‡Phospholipase C Signaling | 0,0014 | 3,38E-02 | 2,121 | ITGA3,RRAS,PLA2G10,HRAS,PLA2G3,PLA2G2A,PRKCZ,PRKCB |
| PPAR Signaling | 0,0117 | 4,08E-02 | -2 | IL1A,RRAS,HRAS,IL1B |
| PPARα/RXRα Activation | 0,0209 | 2,87E-02 | -2,236 | RRAS,HRAS,IL1B,ACVR1C,PRKCB |
| ‡PTEN Signaling | 0,0000 | 6,40E-02 | -2,121 | FOXO4,ITGA3,BAD,RRAS,FGFR4,HRAS,BMPR1B,PRKCZ |
| Rac Signaling | 0,0008 | 4,92E-02 | 2,236 | ITGA3,RRAS,FGFR4,PIK3R6,HRAS,PRKCZ |
| Regulation of Cellular Mechanics by Calpain Protease | 0,0022 | 6,56E-02 | 2 | ITGA3,RRAS,HRAS,EGF |
| *Regulation of eIF4 and p70S6K Signaling | 0,0033 | 3,75E-02 | 2,449 | ITGA3,RRAS,FGFR4,PIK3R6,HRAS,PRKCZ |
| *Renal Cell Carcinoma Signaling | 0,0001 | 6,98E-02 | 2,236 | MET,SLC2A1,RRAS,FGFR4,PIK3R6,HRAS |
| Role of NANOG in Mammalian Embryonic Stem Cell Pluripotency | 0,0000 | 7,14E-02 | 2 | RRAS,FGFR4,PIK3R6,HRAS,BMPR1B,BMP5,APC,WNT5B,WNT5A |
| SAPK/JNK Signaling | 0,0170 | 3,67E-02 | 2 | RRAS,FGFR4,PIK3R6,HRAS |
| ‡STAT3 Pathway | 0,0000 | 8,74E-02 | 3 | CSF2RB,IL1A,IGF1,RRAS,FGFR4,HRAS,IL1B,EGF,BMPR1B |
| Telomerase Signaling | 0,0006 | 5,26E-02 | 2,449 | ELF3,RRAS,FGFR4,PIK3R6,HRAS,EGF |
| Th1 Pathway | 0,0302 | 3,08E-02 | 2 | IFNG,FGFR4,PIK3R6,DLL4 |
| *UVA-Induced MAPK Signaling | 0,0178 | 3,60E-02 | 2 | RRAS,FGFR4,PIK3R6,HRAS |
| ‡VEGF Family Ligand-Receptor Interactions | 0,0000 | 1,17E-01 | 2,714 | RRAS,FGFR4,PLA2G10,PIK3R6,VEGFC,HRAS,PLA2G3,VEGFD,PLA2G2A,PRKCZ,PRKCB |
| ‡VEGF Signaling | 0,0000 | 7,55E-02 | 2,121 | BAD,RRAS,FGFR4,PIK3R6,VEGFC,HRAS,VEGFD,PRKCB |

* Unequivocal linked to non-tumorous disease or different types of cancer

‡ Canonical pathways predicted in TF and TC

**Table S2B: Analysis of significantly regulated canonical pathways (z-score >±2.0 and p<0.05) at TC (cSII/III vs cSI) using IPA predicts a total of 20 pathways of which n=16 are linked to tumour related or immune processes.**

| **Ingenuity Canonical Pathways TC cS23vs1** | **p-value** | **Ratio** | **z-score** | **Molecules** |
| --- | --- | --- | --- | --- |
| ‡CCR3 Signaling in Eosinophils | 4,57E-03 | 3,03E-02 | 2 | HRAS,PLA2G3,PLA2G2A,PRKCZ |
| ‡Endothelin-1 Signaling | 3,02E-03 | 2,60E-02 | 2,236 | HRAS,PLA2G3,NOS3,PLA2G2A,PRKCZ |
| eNOS Signaling | 0,01 | 2,42E-02 | 2 | VEGFC,VEGFD,NOS3,PRKCZ |
| ‡ERK/MAPK Signaling | 3,80E-03 | 2,46E-02 | 2,236 | ITGA3,ELF3,HRAS,PLA2G3,PLA2G2A |
| ‡Fc Epsilon RI Signaling | 3,80E-03 | 3,20E-02 | 2 | HRAS,PLA2G3,PLA2G2A,PRKCZ |
| ‡HGF Signaling | 3,39E-04 | 4,24E-02 | 2 | ITGA3,ELF3,HRAS,IL6,PRKCZ |
| *Huntington's Disease Signaling | 3,72E-02 | 1,62E-02 | 2 | IGF1,HRAS,EGF,PRKCZ |
| ‡IL-8 Signaling | 5,75E-04 | 2,97E-02 | 2,449 | VEGFC,HRAS,VEGFD,EGF,PRKCZ,ITGB3 |
| ‡NF-κB Activation by Viruses | 1,29E-03 | 4,30E-02 | 2 | ITGA3,HRAS,PRKCZ,ITGB3 |
| ‡NF-κB Signaling | 1,45E-02 | 2,17E-02 | 2 | HRAS,EGF,BMPR1B,PRKCZ |
| *Nitric Oxide Signaling in the Cardiovascular System | 2,24E-03 | 3,70E-02 | 2 | VEGFC,VEGFD,NOS3,PRKCZ |
| *‡Ovarian Cancer Signaling | 1,12E-04 | 4,03E-02 | 2 | FGF9,VEGFC,HRAS,VEGFD,EGF,APC |
| ‡Phospholipase C Signaling | 1,32E-03 | 2,53E-02 | 2,449 | ITGA3,NFAT5,HRAS,PLA2G3,PLA2G2A,PRKCZ |
| ‡PI3K/AKT Signaling | 5,13E-04 | 3,88E-02 | 2 | ITGA3,GDF15,HRAS,NOS3,PRKCZ |
| ‡PTEN Signaling | 3,80E-03 | 3,20E-02 | -2 | ITGA3,HRAS,BMPR1B,PRKCZ |
| ‡STAT3 Pathway | 1,82E-04 | 4,85E-02 | 2,236 | CSF2RB,IGF1,HRAS,EGF,BMPR1B |
| *Synaptic Long Term Depression | 2,63E-04 | 3,45E-02 | 2,449 | IGF1,HRAS,PLA2G3,NOS3,PLA2G2A,PRKCZ |
| TGF-β Signaling | 1,29E-03 | 4,30E-02 | 2 | HRAS,BMPR1B,SERPINE1,ACVR1C |
| ‡VEGF Family Ligand-Receptor Interactions | 5,01E-07 | 7,45E-02 | 2,646 | VEGFC,HRAS,PLA2G3,VEGFD,NOS3,PLA2G2A,PRKCZ |
| ‡VEGF Signaling | 2,09E-03 | 3,77E-02 | 2 | VEGFC,HRAS,VEGFD,NOS3 |

* Unequivocal linked to non-tumorous disease or different types of cancer

‡ Canonical pathways predicted in TF and TC

**Table S3**: **List of subset of genes (281 of 687) identified by PANTHER classification related to immune system processes.**

| ADAM8 | CD36 | DLL4 | ICAM1 | KRAS | PIK3R6 | RUNX1 | TDGF1 |
| --- | --- | --- | --- | --- | --- | --- | --- |
| ADAM9 | CD44 | EDN1 | ID2 | LGALS1 | PITX2 | S100A7 | TGFB1 |
| ADAM15 | CD46 | EGF | IFNG | LRG1 | PKM | S100A14 | TGFBR2 |
| ADAM17 | CDC42 | ENPP2 | IGF1 | LY96 | PKN1 | S1PR1 | THBS1 |
| ADD1 | CDH11 | EP300 | IL1A | MAP2K1 | PKNOX1 | SCNN1A | THY1 |
| AKT1 | CEACAM1 | EPAS1 | IL1B | MAP3K7 | PLA2G2A | SDC4 | TIE1 |
| ALDOA | CEACAM5 | EPCAM | IL1RL1 | MAPK1 | PLA2G2D | SELE | TIMP2 |
| ALOX5 | CEACAM6 | EPHA1 | IL1RN | MAPK3 | PLA2G3 | SERINC5 | TLR4 |
| ANGPT1 | CFP | EPHB3 | IL6 | MAPKAPK3 | PLAU | SERPINA1 | TMC6 |
| ANGPT2 | CHAD | EPS8L1 | IL10RA | MCAM | PLAUR | SERPINE1 | TNC |
| ANPEP | CHI3L1 | F11R | IL11 | MED1 | PLCG1 | SERPING1 | TNF |
| ANXA2P2 | CIB1 | FASLG | IL13RA2 | MET | PLCG2 | SETD2 | TNFRSF12A |
| AP1M2 | CLU | FGFR2 | IL15 | MMP1 | PLS1 | SFRP1 | TNFSF10 |
| ATPIF1 | COL1A1 | FGL2 | IL18 | MMP3 | PRF1 | SFRP2 | TNFSF12 |
| BAI1 | COL1A2 | FLI1 | INHBE | MMP9 | PRKCB | SH2B3 | TNFSF13 |
| BAI3 | CREBBP | FLT1 | IRF6 | MMP13 | PRR15L | SHB | TOM1L1 |
| BMP4 | CRIP2 | FN1 | ITGA1 | MRC1 | PTK2 | SIRT1 | UBA52 |
| BMP5 | CRISPLD2 | FST | ITGA3 | MT3 | PTK2B | SLPI | VAMP8 |
| BMPR1A | CST7 | GM-CSF | ITGA5 | MTOR | PTPRB | SMAD3 | VAV2 |
| BMPR1B | CTSG | GPI | ITGA6 | NCAM1 | PTPRC | SMAD5 | VCAM1 |
| C1S | CTSH | GREM1 | ITGA8 | NDRG1 | PTRF | SMOC1 | VEGFA |
| C3 | CTSL | GSN | ITGA9 | NFATC2 | PTX3 | SOD1 | VEGFC |
| C3AR1 | CX3CL1 | HDAC5 | ITGAM | NFKB1 | PXDN | SRC | VIM |
| CAMK2A | CXADR | HIF1A | ITGB1 | NOTCH1 | PYCARD | SRF | VSIG4 |
| CAMK2D | CXCL8 | HIPK1 | ITGB2 | NR4A3 | RAC1 | SRPK2 | VWA2 |
| CAMP | CXCL10 | HIPK2 | ITGB3 | NRXN1 | RAF1 | SSTR2 | WIPF1 |
| CASP8 | CXCL11 | HK2 | ITGB7 | NTRK1 | RB1 | STAB2 | WNT5A |
| CAV1 | CXCL12 | HK3 | ITM2A | OAS1 | RBM47 | STAT1 | ZC3H12A |
| CCL5 | CXCL13 | HKDC1 | JAG1 | PDK1 | RBPJ | STAT3 | ZEB1 |
| CCL8 | CXCL17 | HLA-DPB1 | JAM2 | PDPN | RGCC | SV2B |  |
| CCL11 | CXCR2 | HMOX1 | JAM3 | PECAM1 | RHOA | SYK |  |
| CCL21 | CXCR3 | HOXB3 | JUN | PIK3CA | ROCK1 | TACSTD2 |  |
| CCR2 | CXCR4 | HOXB13 | KCNJ8 | PIK3CD | RORA | TAL1 |  |
| CCR3 | CYBB | HPSE | KDR | PIK3CG | RPS27A | TBX1 |  |
| CD24 | DLC1 | HRAS | KISS1 | PIK3R1 | RPS6KB1 | TBX4 |  |
| CD34 | DLG1 | HSP90B1 | KLK3 | PIK3R2 | RRAS | TCF3 |  |

**Supplemental Table S4: Significantly differentially expressed genes between cSII/III and cSI patients considering the intratumoural compartments of TF and TC.**

|  | **TF of cSII/III vs. cSI** | | **TC of cSII/III vs. cSI** | |
| --- | --- | --- | --- | --- |
| **Genes** | **FC** | **p-value** | **FC** | **p-value** |
| **ANGPT2** | 2.35 | 0.005 | 1.84 | 0.085 |
| **BAI1** | 2.03 | 0.035 | 1.39 | 0.342 |
| **BAI3** | 4.7 | 0.003 | 2.89 | 0.039 |
| **BMP5** | 4.52 | 0.003 | 2.93 | 0.046 |
| **BMPR1B** | 2.34 | 0.010 | 2.5 | 0.023 |
| **CAMK2A** | 2.46 | 0.110 | 2.65 | 0.029 |
| **CCL11** | 2.93 | 0.033 | 1.14 | 0.804 |
| **CDH11** | 1.49 | 0.043 | 2.17 | 0.030 |
| **CEACAM1** | 3.21 | 0.003 | 3.31 | 0.002 |
| **CEACAM5** | 3.04 | 0.022 | 2.27 | 0.126 |
| **CHAD** | 3.95 | 0.005 | 1.97 | 0.177 |
| **CTSG** | 3.24 | 0.027 | 1.93 | 0.233 |
| **CX3CL1** | 3.82 | 0.002 | 3.5 | 0.005 |
| **CXCL17** | 3.21 | 0.020 | 2.17 | 0.126 |
| **DLL4** | 2.34 | 0.008 | 2.11 | 0.017 |
| **EGF** | 4.43 | 0.002 | 3.76 | 0.005 |
| **EPHA1** | 2.45 | 0.010 | 2.01 | 0.021 |
| **EPS8L1** | 2.75 | 0.001 | 2.3 | 0.008 |
| **FST** | 4.24 | 0.006 | 3.24 | 0.021 |
| **GM-CSF** | 2.21 | 0.037 | 1.99 | 0.047 |
| **GREM1** | 3.31 | 0.017 | 1.51 | 0.368 |
| **HKDC1** | 2.16 | 0.007 | 1.34 | 0.345 |
| **HOXB13** | 3.26 | 0.019 | 2.48 | 0.073 |
| **IFNG** | 2.0 | 0.035 | 1.78 | 0.100 |
| **IGF1** | 2.27 | 0.015 | 2.1 | 0.047 |
| **IL1A** | 3.52 | 0.019 | 2.2 | 0.117 |
| **IL1B** | 2.27 | 0.007 | 1.8 | 0.111 |
| **IL6** | 2.38 | 0.054 | 4.9 | 0.003 |
| **IL13RA2** | 2.87 | 0.037 | 2.31 | 0.110 |
| **IL15** | 2.05 | 0.038 | 1.73 | 0.107 |
| **INHBE** | 3.33 | 0.020 | 2.07 | 0.143 |
| **ITGA3** | 2.32 | 0.002 | 2.16 | 0.005 |
| **ITGA8** | 2.08 | 0.135 | 3.28 | 0.009 |
| **ITGB3** | 5.3 | <0.001 | 5.71 | <0.001 |
| **KISS1** | 3.3 | 0.019 | 2.24 | 0.131 |
| **KLK3** | 2.9 | 0.040 | 1.9 | 0.241 |
| **LRG1** | 3.31 | 0.024 | 2.8 | 0.054 |
| **MCAM** | 2.14 | 0.013 | 1.95 | 0.024 |
| **MET** | 2.62 | 0.004 | 1.92 | 0.065 |
| **MMP1** | 3.91 | 0.024 | 1.47 | 0.471 |
| **MMP3** | 3.36 | 0.028 | 2.07 | 0.138 |
| **MMP13** | 3.39 | 0.009 | 2.5 | 0.087 |
| **MT3** | 5.68 | <0.001 | 2.26 | 0.131 |
| **NRXN1** | 2.32 | 0.019 | 1.6 | 0.161 |
| **PIK3R6** | 2.54 | 0.006 | 2.04 | 0.060 |
| **PITX2** | 2.96 | 0.025 | 1.8 | 0.222 |
| **PKNOX1** | 2.17 | 0.004 | 1.78 | 0.031 |
| **PLA2G2A** | 2.91 | 0.023 | 2.69 | 0.039 |
| **PLA2G3** | 4.64 | 0.002 | 3.37 | 0.014 |
| **PLS1** | 3.84 | 0.003 | 2.63 | 0.013 |
| **PRR15L** | 3.24 | 0.021 | 2.43 | 0.031 |
| **PTPRB** | 3.02 | 0.003 | 3.19 | 0.003 |
| **PTRF** | 2.07 | 0.005 | 1.79 | 0.029 |
| **PTX3** | 3.49 | 0.045 | 2.93 | 0.040 |
| **RRAS** | 2.46 | 0.037 | 2.24 | 0.101 |
| **S1PRI1** | 1.72 | 0.081 | 2.35 | 0.037 |
| **SCNN1A** | 3.05 | 0.007 | 2.3 | 0.054 |
| **SERPINE1** | 2.01 | 0.013 | 2.36 | 0.012 |
| **SFRP1** | 2.48 | 0.039 | 1.84 | 0.21 |
| **SHB** | 2.77 | 0.010 | 2.22 | 0.023 |
| **SLPI** | 5.69 | 0.002 | 2.4 | 0.102 |
| **SMOC1** | 2.56 | 0.017 | 1.79 | 0.091 |
| **SSTR2** | 3.03 | 0.020 | 2.22 | 0.131 |
| **STAB2** | 3.3 | 0.016 | 2.23 | 0.108 |
| **SV2B** | 2.45 | 0.005 | 1.86 | 0.067 |
| **TACSTD2** | 2.83 | 0.005 | 2.1 | 0.088 |
| **TBX4** | 3.57 | 0.016 | 1.66 | 0.371 |
| **TDGF1** | 2.23 | 0.044 | 1.83 | 0.093 |
| **TIE1** | 2 | 0.034 | 2.42 | 0.033 |
| **TNC** | 2.87 | 0.013 | 2.02 | 0.138 |
| **TNFRSF12A** | 2.81 | 0.021 | 2.19 | 0.117 |
| **TOM1L1** | 1.53 | 0.347 | 2 | 0.022 |
| **VEGFC** | 4.73 | 0.004 | 3.61 | 0.004 |
| **VWA2** | 4.58 | 0.004 | 2.05 | 0.193 |
| **WNT5A** | 2.53 | 0.026 | 2.27 | 0.051 |

*Note: TF = Tumour Front, TC = Tumour Centre, cS = clinical Stage, FC = Fold Change, FC >±2.0 and p value <0.05*

**Supplemental Table S5: Comparison of IL6 staining intensities between the groups of cSI compared to cSII/III.**

| **Expression** | **Clinical Stage** | n (evaluable Patients) | **Staining Intensity** | | | | **p-value** |
| --- | --- | --- | --- | --- | --- | --- | --- |
|  |  |  | 0 | 1 | 2 | 3 |  |
|  |  |  | % (n) | % (n) | % (n) | % (n) |  |
| **I**L6 | 1 | 18 | - | - | 11.1 (2) | 88.9 (16) | 0.232 |
|  | 2 / 3 | 12 | - | - | - | 100.0 (12) |  |

**Supplemental Table S6: Comparison of TILs between the groups of cSI compared to cSII/III.**

| **Expression** | **Clinical Stage** | n (evaluable Patients) | **TILs** | | | **p-value** |
| --- | --- | --- | --- | --- | --- | --- |
|  |  |  | 0-10%  % (n) | 20-40%  % (n) | 50-90%  % (n) |  |
| **T**ILs | 1 | 21 | 4.8 (1) | 52.4 (11) | 42.9 (9) | **0.003** |
|  | 2 / 3 | 14 | 50.0 (7) | 42.9 (6) | 7.1 (1) |  |

**Figure S1: IPA predictions in comparison analysis between TF versus TC groups.** IPA comparison analysis shows a selection of relevant different disease related and biological functions in cSII/III vs. cSI patients of TF and TC groups. The heat map is based on the activation z-score ± 2.0, " activated " (orange) or " inhibited " (blue) function respectively.


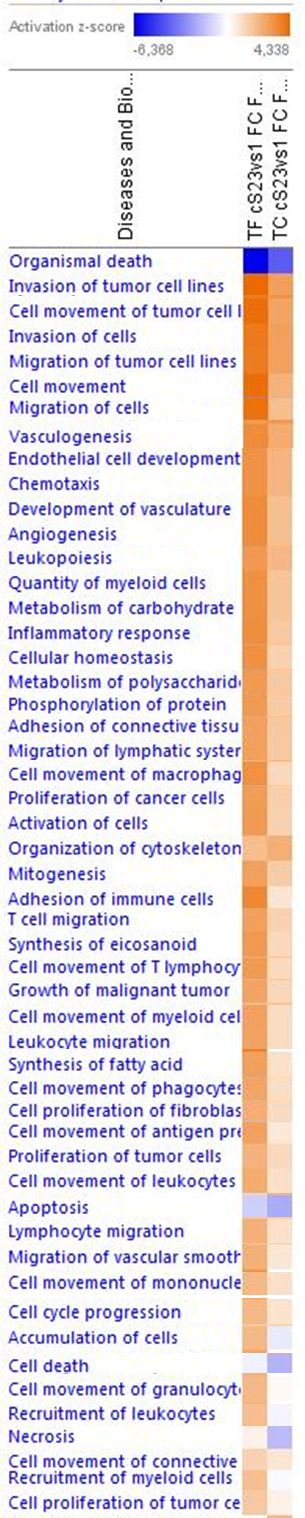


**Figure S2: Immunostaining of IL6 comparing tumours of cSI and cSII/III patients.**

TF of cSI seminoma patients (left column) and cSII/III patients (right column) showing no significant different IL-6 expression patterns (p=0.232) at a magnification of x100.


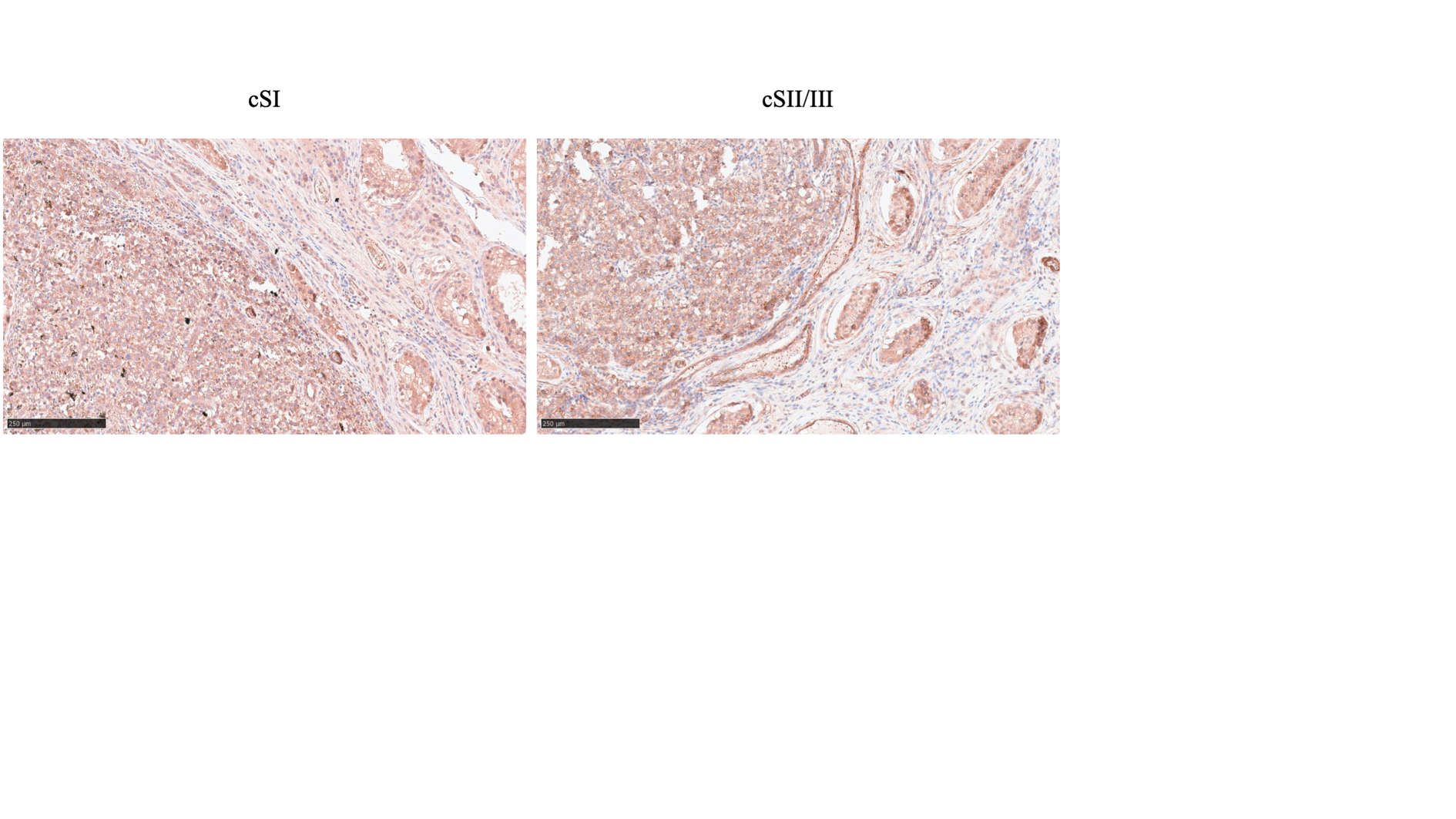


**Figure S3: Assessment of TILs in cSI and cSII/III seminoma tumours.**

TF of cSI seminoma patients (left column) shows significant more TILs compared to cSII/III patients (right column) (p=0.003) at a magnification of x100.


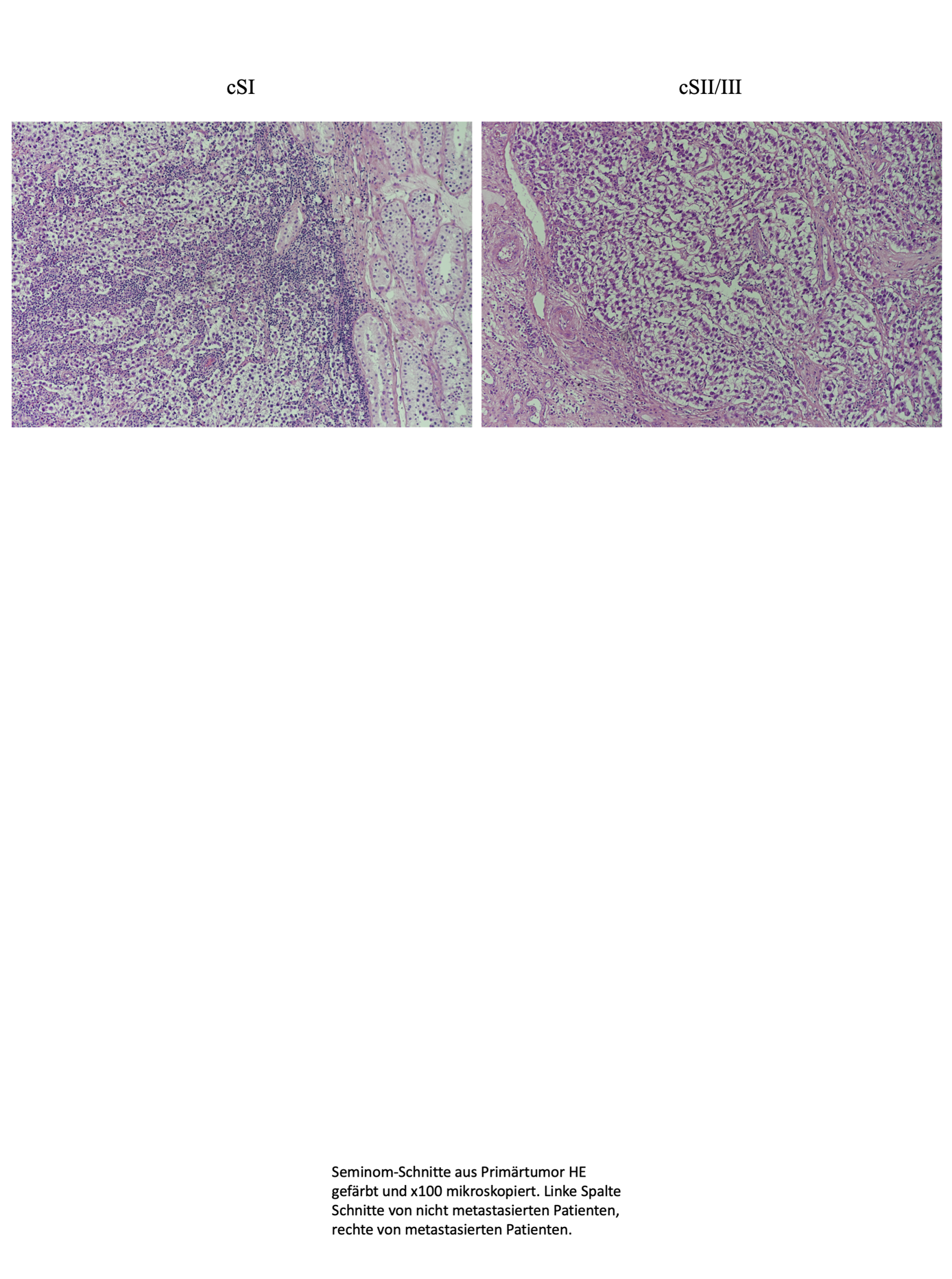

Supplement: Supplementary file 2 — SUPPLEMENTAL MATERIAL [file 41416_2021_1621_MOESM2_ESM.docx]
